# Intranasal Salvinorin A Improves Long-term Neurological Function via Immunomodulation in a Mouse Ischemic Stroke Model

**DOI:** 10.1007/s11481-021-10025-4

**Published:** 2021-10-01

**Authors:** Dilidaer Misilimu, Wei Li, Di Chen, Pengju Wei, Yichen Huang, Sicheng Li, John Grothusen, Yanqin Gao

**Affiliations:** 1 State Key Laboratory of Medical Neurobiology, MOE Frontier Center for Brain Science and Institutes of Brain Science, Fudan University, Shanghai, China; 2 Department of Anesthesiology and Critical Care, Perelman School of Medicine at the University of Pennsylvania, Philadelphia, PA19104 USA

**Keywords:** Salvinorin A, Neutrophils, Microglia/macrophage, Blood–brain barrier, White matter injury, Ischemic stroke

## Abstract

Salvinorin A (SA), a highly selective kappa opioid receptor agonist, has been shown to reduce brain infarct volume and improve neurological function after ischemic stroke. However, the underlying mechanisms have not been fully understood yet. Therefore, we explored whether SA provides neuroprotective effects by regulating the immune response after ischemic stroke both in the central nervous system (CNS) and peripheral circulation. In this study, adult male mice were subjected to transient Middle Cerebral Artery Occlusion (tMCAO) and then were treated intranasally with SA (50 μg/kg) or with the vehicle dimethyl sulfoxide (DMSO). Multiple behavioral tests were used to evaluate neurofunction. Flow cytometry and immunofluorescence staining were used to evaluate the infiltration of peripheral immune cells into the brain. The tracer cadaverine and endogenous immunoglobulin G (IgG) extravasation were used to detect blood brain barrier leakage. We observed that SA intranasal administration after ischemic stroke decreased the expression of pro-inflammatory factors in the brain. SA promoted the polarization of microglia/macrophages into a transitional phenotype and decreased the pro-inflammatory phenotype in the brain after tMCAO. Interestingly, SA treatment scarcely altered the number of peripheral immune cells but decreased the macrophage and neutrophil infiltration into the brain at 24 h after tMCAO. Furthermore, SA treatment also preserved BBB integrity, reduced long-term brain atrophy and white matter injury, as well as improved the long-term neurofunctional outcome in mice. In this study, intranasal administration of SA improved long-term neurological function via immuno-modulation and by preserving blood–brain barrier integrity in a mouse ischemic stroke model, suggesting that SA could potentially serve as an alternative treatment strategy for ischemic stroke.

**Graphic Abstract:**

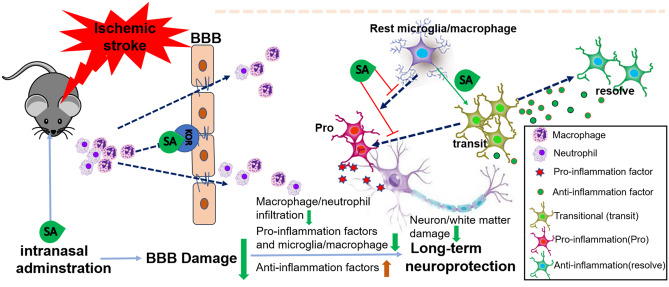

**Supplementary Information:**

The online version contains supplementary material available at 10.1007/s11481-021-10025-4.

## Introduction

As the leading cause of death and permanent morbidity worldwide, stroke accounts for 5.5 million deaths and 5 million disabilities annually (Gorelick 2019). Ischemic stroke accounts for 80% of stroke cases and still lacks therapeutic choices for effective treatment strategies for the majority of stroke patients (Lalu et al. 2020). Intravenous alteplase and endovascular thrombectomy are effective rescue treatments for stroke patients (Petty et al. 2021) but unfortunately only a limited number of patients benefit from this reperfusion therapy because of the narrow therapeutic window and contraindications (Bhaskar et al. 2018). Currently there are clinical trials employing neuroprotectants that target early pathogenic mechanisms in stroke (Shafie and Yu 2021). If successful, these agents could rescue more stroke patients and improve long-term neurological outcome.

Immunity and inflammation play a pivotal role in the pathological progression of ischemic stroke, including acute events and long-term prognosis of stroke (Jayaraj et al. 2019). After ischemic stroke, the compromised blood–brain barrier (BBB) allows peripheral immune cells, including neutrophils and macrophages, to leak into the extracellular space of the brain parenchyma and promote the progression of brain injury (Jian et al. 2019). The peripheral immune cells that infiltrate into the brain exacerbate the breakdown of the BBB (Jiang et al. 2018) and promote microglia/macrophages to polarize toward a pro-inflammatory phenotype (Yunna et al. 2020; Lyu et al. 2021). As the first leukocyte subset to appear in the ischemic brain, neutrophils damage the BBB by releasing reactive oxygen species (ROS) and proteolytic enzymes (Malone et al. 2019), resulting in severe endothelial damage, destruction of adjacent blood vessels, and in some cases hemorrhagic transformation (Perez-de-Puig et al. 2015). Evidence has been reported that immunomodulators targeted at the peripheral immune system contribute to neuroprotective effects and improve functional outcomes after ischemic stroke (Chen et al. 2013). In addition, the restoration of BBB following disruption is involved in the underlying mechanism of the neuroprotective effect by alleviating the peripheral inflammation (Chen et al. 2013).

Salvinorin A (SA) is a highly selective non-opioid kappa opioid receptor (KOR) agonist. It is an active component from Salvia divinorum that is a perennial herb and has been consumed by humans for several centuries (Grundmann et al. 2007) without producing frank hallucinatory or dysphoric effects, which is different from other KOR agonists (Chunhua et al. 2014). SA has side effects of diuresis, hypothermia, reduced brain metabolism, and psychotropic effects, which could potentially all contribute to protective effects in the setting of stroke (Grothusen 2021). Previous studies have shown the safety of SA administration both in mice and human (Cunningham et al. 2011). Mark Mowry et al. investigated the acute physiologic and chronic histologic changes in rats and mice exposed to salvinorin A, and the data shows that no effects were seen on cardiac conduction, temperature, or pulse pressure (Mowry et al. 2003).Consistent with results from nonhuman animal research, many human trials have shown Salvinorin A inhalation produced no significant changes in heart rate, blood pressure, O_2_ saturation and core temperature suggesting a safe physiological profile of SA (Johnson et al. 2011; Mendelson et al. 2011). A recent study demonstrating that intranasal SA improves neurological outcome in a rhesus monkey autologous clot ischemic stroke model is very encouraging (Wu et al. 2020). Further studies are needed to evaluate mechanisms that were not investigated in the past and also the role of SA treatment in immune-regulation after ischemic stroke.

In this study, we tested the hypothesis that SA administration reduces brain tissue injury and improves long-term neurological recovery after ischemic stroke by reducing the infiltration of peripheral inflammatory cells into the ischemic brain and by protecting the integrity of the BBB.

## Methods and Materials

### Animals and Experimental Study Design

Adult male C57BL/6 J mice (8–10 weeks old) were purchased from Shanghai Research Center for Model Organisms Co., Ltd (Shanghai, China). All the animal experiments were conducted in strict accordance with the National Institutes of Health Guide for the Care and Use of Laboratory Animals and ARRIVE (Animal Research: Reporting in Vivo Experiments) guidelines. Experimental protocols were approved by the Institutional Animal Care and Use Committee at Fudan University. Adequate measures were taken to minimize the number of animals used and to minimize animal suffering. Mice were housed in an individually ventilated facility with a temperature-controlled environment under 12 h light/12 h darkness cycle, and all animals were allowed free access to food and water. Animals were randomly divided into the following groups: (1) Sham group with or without SA: mice in this group had the carotid arteries exposed without transient Middle Cerebral Artery Occlusion (tMCAO), mice received intranasal administration of 10% DMSO (vehicle) or SA (50 μg/kg body weight) dissolved in 10%DMSO; (2) tMCAO + Veh group: tMCAO mice received intranasal administration of 10% DMSO (vehicle); (3) tMCAO + SA group: tMCAO mice received intranasal administration of SA (50 μg/kg bodyweight) dissolved in 10%DMSO.

### Intranasal Administration of Salvinorin A

Mice were administered SA or DMSO under isoflurane anesthesia once every 2 days for 6 consecutive days after tMCAO. The first dose was given 10 min after the reperfusion, the subsequent doses were administered at similar time intervals after the surgical procedure under anesthesia for a given animal. Briefly, five drops (2 μl/drop) of SA or DMSO were applied alternately into each nostril with a 2 min interval between drops for total of 10 min for each treatment.

### tMCAO Model

tMCAO model was accomplished by intraluminal occlusion of the left middle cerebral artery for 60 min as previously described (Shi et al. 2016). Sham-operated animals underwent anesthesia and surgical exposure of the arteries without MCAO induction. Rectal temperature was maintained at 37 ± 0.5 °C during surgery with a temperature-controlled heating pad. Regional cerebral blood flow (rCBF) was measured using laser speckle during the entire procedure. Animals that did not show an rCBF reduction of at least 75% of the baseline levels or that died after ischemia induction were excluded from further experimentation. Surgeries and all measurements were performed by investigators blinded to mouse genotype and experimental group assignments.

### Flow Cytometry

At 24 h after tMCAO, mouse blood was collected from heart puncture after deep anesthesia. The blood and spleen were collected for flow cytometry, which was performed as described in the Supplemental methods material.

### Western Blot

Equal amounts of cell lysate from brain samples were subjected to western blot analysis, which was performed as previously described (Zhang et al. 2016). Protein concentration was measured using the BCA kit (Thermo Fisher Scientific, USA). Primary antibodies were rabbit monoclonal anti-KOR (1:1000, ab183825, Abcam), rabbit anti-MMP-2 (1:1000, ab97779, Abcam), and rabbit anti-MMP-9 (1:1000, ab38898, Abcam). Horseradish peroxidase-conjugated (1:1000, 7074S, CST) anti-rabbit antibody was used as secondary antibody. Blots were imaged using ChemiDoc MP (Bio-Rad).

### Assessment of BBB Impairment

After tMCAO, BBB permeability was assessed by measuring the extravasation of an intravenously- injected fluorescent tracer Alexa 555 cadaverine (0.95KDa, Invitrogen, 200 mg/mouse), and endogenous plasma IgG into the brain parenchyma. Coronal brain sections were processed for direct fluorescence detection of Alexa 555, or subjected to immunofluorescent labeling of IgG and detection. The brain volume with cadaverine or IgG leakage was calculated on six equally spaced brain sections encompassing the MCA territory (Shi et al. 2016).

### Quantitative Real-Time PCR

The cytokines and inflammatory factors, such as IL-1β, TNF-α, TNF-β, IL-10, CCL-2 and CCL-3 were measured by Real-time PCR. Real-time PCR was performed on an Opticon 2 Real-Time PCR Detection System (BioRad, Hercules, CA, USA) with the primers and SYBR green PCR Master Mix (Applied Biosystems, Waltham, USA) to measure the inflammatory factors in brain tissue. The expression level of mRNAs was calculated as fold changes versus sham. All reactions were performed in triplicate. The primer sequences are in the Supplementary Materials.

### Gelatin Zymography

Polyacrylamide gels containing 8% sodium dodecyl sulfate (SDS) and 0.1% gelatin were used to carry out zymography. The protein samples (20 μg) from brain tissue were added to 2 × non-reducing sample buffer. Electrophoresis in a mini-gel apparatus (Bio-Rad, Hercules, USA) was performed. The gels were washed with 2.5% Triton X-100 for 1 h to remove the SDS and incubated for 40 h at 37 °C in digestion buffer. After incubation, gels were stained for 3 h with 0.5% coomassie blue and de-stained in buffer containing 30% methanol and 10% glacial acetic acid. Quantity One software (Bio-Rad, Hercules, USA) was used for scanning and analyzing bands reflecting gelatinolytic activity. Three independent experiments were performed in duplicate.

### Immunohistochemical Staining

Immunohistochemical staining was performed as previously described (Miao et al. 2020). Briefly, coronal brain sections were blocked with 5% goat serum in phosphate-buffered saline with 0.1% Triton-X 100 for 1 h, followed by primary antibody incubations for 1 h at room temperature and overnight incubation at 4 °C. The following primary antibodies were used: Rat anti MAP2 (1:400; Millipore), Rabbit anti Ly6G (1:300; Abcam), Rabbit anti F4/80 (1:300; Bio Legend), Alexa Flour 488-conjugated (1:300; Invitrogen), Rat anti CD31 (1:300; BD Biosciences). Rabbit anti-MMP-2 (1:200, ab97779, Abcam), goat anti-CD206 (1:250, AF2535, R&D)**,** rat anti-CD16/32 (1:250, ab25235, Abcam), and rabbit anti-Iba1 (1:1000, ab5076, Abcam).

### Neurological Outcome Assessments

An adhesive removal test was performed to evaluate sensory and motor deficits (Bouet et al. 2009). The tests are evaluated before tMCAO and at 3, 5, 7, 14, and 21 days after tMCAO. The corner test was used for detecting long-term sensorimotor dysfunction after tMCAO (Zhang et al. 2019a). The rotarod test was used to assess motor performance by measuring the time of latency to remain on an elevated rotating accelerating rod (4 to 40 r/min over 300 s) (Zhang et al. 2019a). The corner and rotarod tests are evaluated before tMCAO and at 3, 5, 7, 14, 21, 28, and 35 days after tMCAO. The Morris water maze test was performed to assess the spatial learning and memory (Zhang et al. 2019a; Moreira-de-Sá et al. 2020). Morris water maze tests were performed at 30 to 35 days after tMCAO. Details of these neurological outcome assessments are presented in the supplemental data. All the tests were scored by an investigator who was blind to the experimental design to avoid bias.

### Statistical Analysis

All data are presented as mean ± SEM. One-way analysis of covariance (ANOVA, Tukey) was used to analyse the 4 groups for flow cytometry. For qRCR, WB and immunostaining result, there is no significant change between the sham group and sham + SA group, so we combine the two groups and one-way analysis of covariance (ANOVA, Tukey) was also used for analysis; Two-way analysis of covariance was used to study the difference in neurological and cognitive functional tests over time, and unpaired 2-tailed student t test was performed to compare 2 groups in Graph Pad Prism 7 (Graph Pad Software Inc.). A P value < 0.05 was considered statistically significant.

## Results

### Intranasal Administration of SA Increased the Expression of KOR on the Endothelium Cells of Brain in Tmcao Mice

**Fig. 1:**
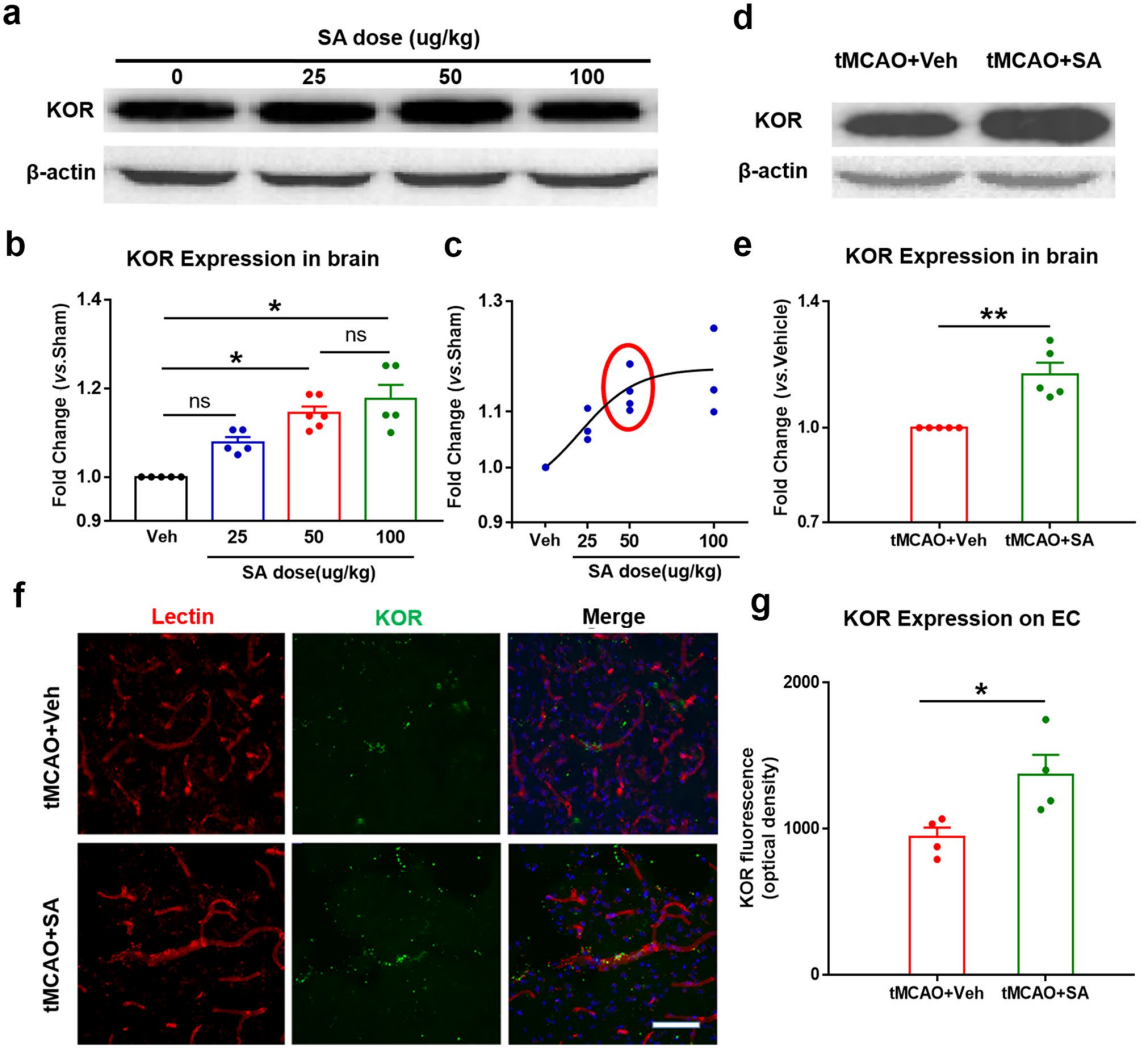
SA treatment increased the expression of KOR protein in brain tissue after tMCAO. **a** The expression of total KOR was examined by western blot after different SA doses in normal mice. **b** Quantification of the KOR expression in the brain. n = 5–6/group. **c** The graph of different SA doses and KOR expression. When the SA dose is increaded beyond 50 µg/kg, the KOR expression stays constant. **d** The expression of total KOR was examined by western blot in tMCAO mice with vehicle or SA treated. n = 5 mice/group. **e** Quantification of the KOR expression in the brain of tMCAO mice. **f** Representative image of KOR expression on endothelial cell markers (lectin). n = 4 mice/group. Scale bar: 40 μm. All data are presented as mean ± SEM. *p ≤ 0.05, *ns*: no significant, as indicated

It has been reported that the dose of SA and the volume of cerebral infarction are negatively correlated within an initial concentration range in mice that suffered tMCAO, and the infarct volume is most significantly decreased when the SA dose is 50 μg/kg (Chen et al. 2016). In order to confirm the relationship between the therapeutic concentration of SA and the expression of KOR in the brain, we tested four different doses of SA: 0 μg/kg, 25 μg/kg, 50 μg/kg and 100 μg/kg in normal mice (10–12 weeks aged, male). Western blot was used to quantitate the expression of KOR in brain tissue at 24 h after SA administration. The result showed that the expression of KOR was positively correlated with the SA dose. Administering a dose of 50 µg/kg, SA significantly increased the expression of KOR compared to the non-administration group. A dose of 100 µg/kg SA did not significantly increase KOR expression compared to the 50 µg/kg dose group (Fig. 1a-c). We choose 50 µg/kg as the administration dose in this study. In addition, we tested the KOR expression in the tMCAO mice after SA treatment. We administered SA to the mice at a dose of 50 µg/kg 10 min following ischemic stroke. Western blot was performed to detect KOR content in the brain tissue around the injury area at 24 h after tMCAO. The result showed that the level of KOR expression significantly increased (Fig. 1d-e). KOR is expressed on a number of hematopoietic cell populations, leukocytes and brain cells (Rogers 2021). The immunofluorescence of KOR/Lectin double staining showed that the expression of KOR on endothelial cells significantly increased in the tMCAO + SA group compared to the tMCAO + Veh group. Taken together, our results suggested that 50 µg/kg intranasal administration was an appropriate dose, which can increase the expression of KOR in brain tissue, including vascular endothelial cells.

### SA Treatment Inhibited the Expression of Pro-inflammatory Factors and Cell Cytokines as well as Decreased the Pro-inflammatory Microglia/Macrophages in the Brain after Tmcao

**Fig. 2:**
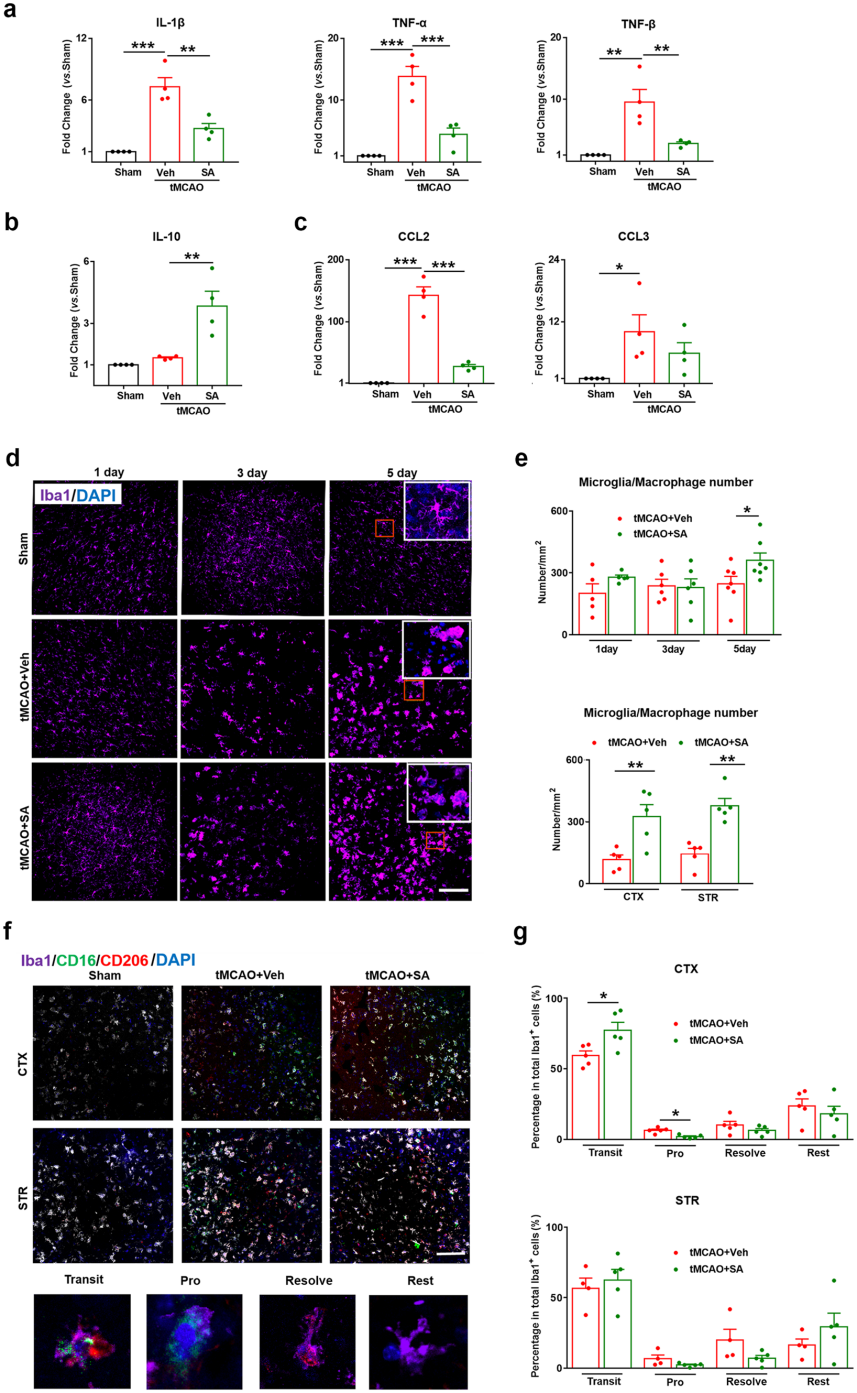
SA treatment inhibited the expression of pro-inflammatory factors and cell cytokines as well as decreased the pro-inflammatory microglia/macrophages in the brain after tMCAO. **a** Pro-inflammation markers (IL-1β, TNF-α and TNF-β) were measured in the ipsilateral hemisphere at 24 h after tMCAO. n = 3–4 mice/group. **b** Measurement of anti-inflammation markers IL-10. n = 4 mice/group. **c** Measurement of chemokines markers CCL2 and CCL3. n = 4 mice/group. **d** Representative images from the inner border of infarction in the cortex (CTX) and corpus striatum (STR) at 1,3,5 days after tMCAO, showing immunofluorescence for the following markers: Iba1 (purple, microglia/macrophage), DAPI (blue, nuclear marker). Scale bar, 40 µm. **e** Quantification for Iba1 staining. n = 5–7 mice/group. **f** Representative images of immunofluorescence for different phenotypes of microglia/macrophage with showing following markers: Iba1 (purple, microglia/macrophage), CD16 (green, activated pro-inflammatory microglia/macrophage), CD206 (red, activated anti-inflammatory microglia/macrophage), DAPI (blue, nuclear marker). The Iba1^+^/CD16^+^ cells are considered as pro-inflammatory microglia/macrophages (pro), Iba1^+^/CD206^+^ cells are anti-inflammatory microglia/macrophages (resolve), Iba1^+^/CD16^+^/CD206^+^ cells are the transitional phenotype (transit) and only Iba1^+^ cells are under quiescent condition (rest). Scale bar, 40 µm. **g** Quantification for Iba1staining. n = 4–5 mice/group. All data are presented as mean ± SEM. *p ≤ 0.05, **p ≤ 0.01, ***p ≤ 0.001, as indicated

Previous study reported that KOR agonists exhibited potent anti-inflammatory capacities in neuroinflammatory disorders (Tangherlini et al. 2019). The KOR agonist nalfurafine has been proved to reduce neuroinflammation and drive remyelination in models of CNS demyelinating disease (Denny et al. 2021). The release of inflammatory factors starts in the early stages after tMCAO. To investigate whether SA reduces the inflammatory and chemokine levels in the post-ischemic brain, we measured a panel of inflammatory mediators in the ipsilateral brain hemisphere at 24 h after tMCAO using qPCR. The expression of pro-inflammatory factors (IL-1β, TNF-α and TNF-β) and inflammatory cell chemokines (CCL2 and CCL3) in the brain were found to be significantly increased in brain after tMCAO compared to the sham group. When compared with the tMCAO + Veh group, the tMCAO + SA group showed a significantly reduced expression level of the pro-inflammatory factor (Fig. 2a) and chemokine CCL2 (Fig. 2c) in the brain. SA treatment after tMCAO increased the anti-inflammatory factor IL-10 (Fig. 2b). In short, SA treatment reduced the levels of pro-inflammatory factors and chemokines and promoted the expression of anti-inflammatory factors at the early stage of ischemic stroke.

Microglia/macrophages can be activated rapidly after ischemic brain injury (Deng et al. 2020) and classified as resting phenotype (Iba1^+^/CD16^−^/CD206^−^), pro-inflammatory phenotype (Iba1^+^/CD16^+^/CD206^−^), anti-inflammatory phenotype (Iba1^+^/CD16^−^/CD206^+^) and transitional phenotype (Iba1^+^/CD16^+^/CD206^+^) by immunofluorescence staining (Ma et al. 2017). We harvested brain tissue sections at 1, 3, and 5 days after tMCAO and used immunofluorescence to detect the activated and polarization of microglia/macrophages. The results showed that there was no difference between the activation interactions of the microglia/macrophages at 1 and 3 days after tMCAO. However, on day 5 after tMCAO, the number of microglia/macrophages in the tMCAO + SA group significantly increased compared to the tMCAO + Veh group and the number of cell activations in the striatum and cortex were both significantly higher than that of tMCAO + Veh group (Fig. 2d-e). To further explore the phenotype of microglia/macrophages, we employed the immunofluorescence staining on the 5th day after tMCAO, it was observed that the proportion of microglia/macrophages which present transitional phenotypes (transit) in the SA treatment group was significantly increased while pro-inflammatory phenotype (pro) was significantly decreased when compared with the tMCAO + Veh group in the CTX instead of STR of the brain. There was no difference between the groups when quantifying the anti-inflammatory (resolve) and resting (rest) phenotypes of microglia/macrophages. These results suggest that SA treatment inhibited the expression of pro-inflammatory factors and cell cytokines as well as decreased the pro-inflammatory microglia/macrophages in the brain after tMCAO.

### SA Treatment Attenuated the Infiltration of Peripheral Immune Cells into the Brain after tMCAO at 24 h after tMCAO

**Fig. 3:**
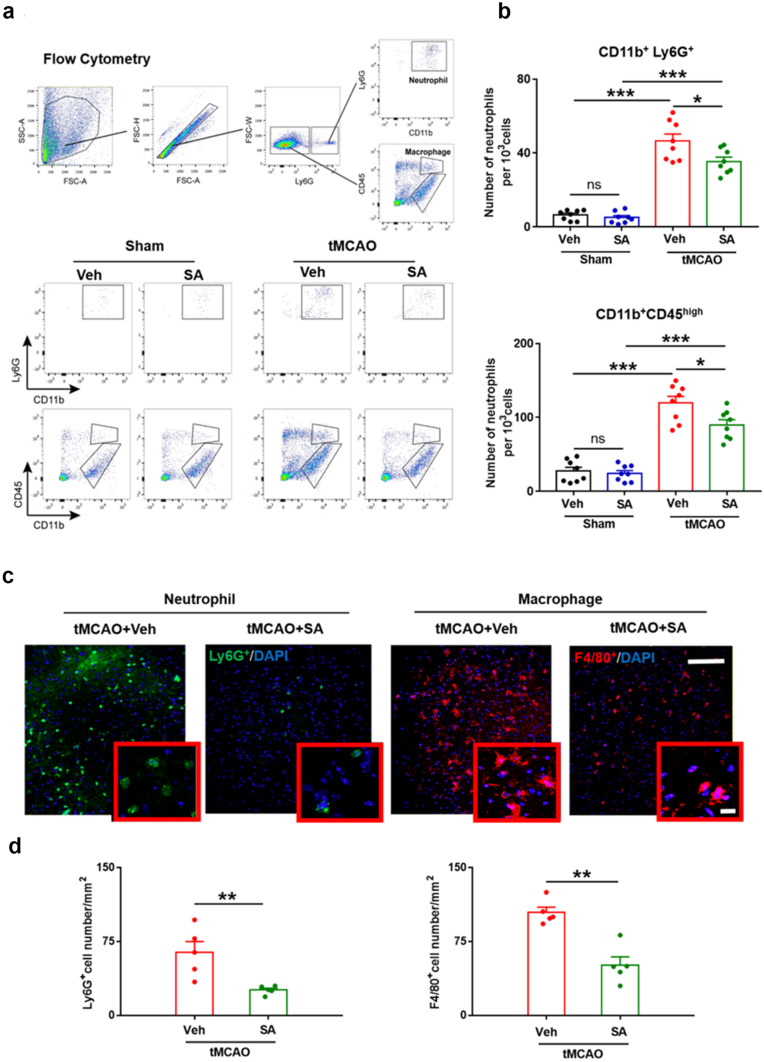
SA treatment attenuated the infiltration of peripheral immune cells after tMCAO. Cell suspensions were prepared from the ipsilateral hemispheres and flow cytometry was performed to quantify Ly6G^+^ (infiltrated neutrophils) and CD45^high^ (macrophages and activated microglia) cells among the CD11b^+^ cell populations. **a** Representative FACS analyses of neutrophils (CD11b^+^Ly6G^+^) and macrophages and activated microglia (CD11b^+^Ly6G^−^CD45^high^) (Upper panel). Representative FACS for numbers of neutrophils and macrophages in different groups (Lower panel). **b** Quantification of neutrophils (upper panel) and macrophages (lower panel). n = 8 mice/group. **c** Representative images of immunostaining for infiltrated neutrophils (Ly6G^+^) and macrophages (F4/80^+^). Scale bar, 40 µm. **d** Quantification for immunostaining. n = 5 mice/group. All data are presented as mean ± SEM. *p ≤ 0.05, ** *p* ≤ 0.01, ***p ≤ 0.001, as indicated

Blood neutrophils and macrophages infiltrating into the brain play a critical role in the development of brain parenchyma injury and neurological damage after tMCAO. To test whether SA treatment post-tMCAO attenuates the infiltration of peripheral immune cells into brain, we performed a flow cytometry-based strategy to quantitatively analyze the brain infiltration of CD11b^+^Ly6G^+^ (representing neutrophils) and Ly6G^−^CD11b^+^CD45^high^ (representing macrophages and activated microglia) cells among the CD11b^+^ cell populations at 24 h after tMCAO (Fig. 3). In sham groups, SA treatment didn’t change the number of infiltrated neutrophils or macrophages and activated microglia. CD11b^+^Ly6G^+^ cells were dramatically increased in the post-tMCAO brain hemisphere compared with sham-operated mice (Fig. 3a-b), reflecting the robust infiltration of blood neutrophils into the brain. In the Ly6G^−^ cell population, tMCAO also induced an increase in the number of CD11b^+^CD45^high^ cells (Fig. 3a-b). In contrast to tMCAO + Veh mice, tMCAO + SA group mice showed significantly reduced neutrophils and microglia/ macrophages infiltrating into the brain.

These results were confirmed by immunohistochemistry in another cohort of mice, in which neutrophils and macrophages were immunolabeled by Ly6G and F4/80, respectively (Fig. 3c). Quantitatively, the number of Ly6G^+^ and F4/80^+^ cells that infiltrated into brain was significantly smaller in tMCAO + SA mice compared to tMCAO + Veh mice, both in CTX and STR (Fig. 3d). Together, the results show that SA treatment significantly reduced the infiltration of peripheral immune cells into the brain after tMCAO.

### SA Administration did not Change the Number of Peripheral Neutrophils and Macrophages after tMCAO

**Fig. 4:**
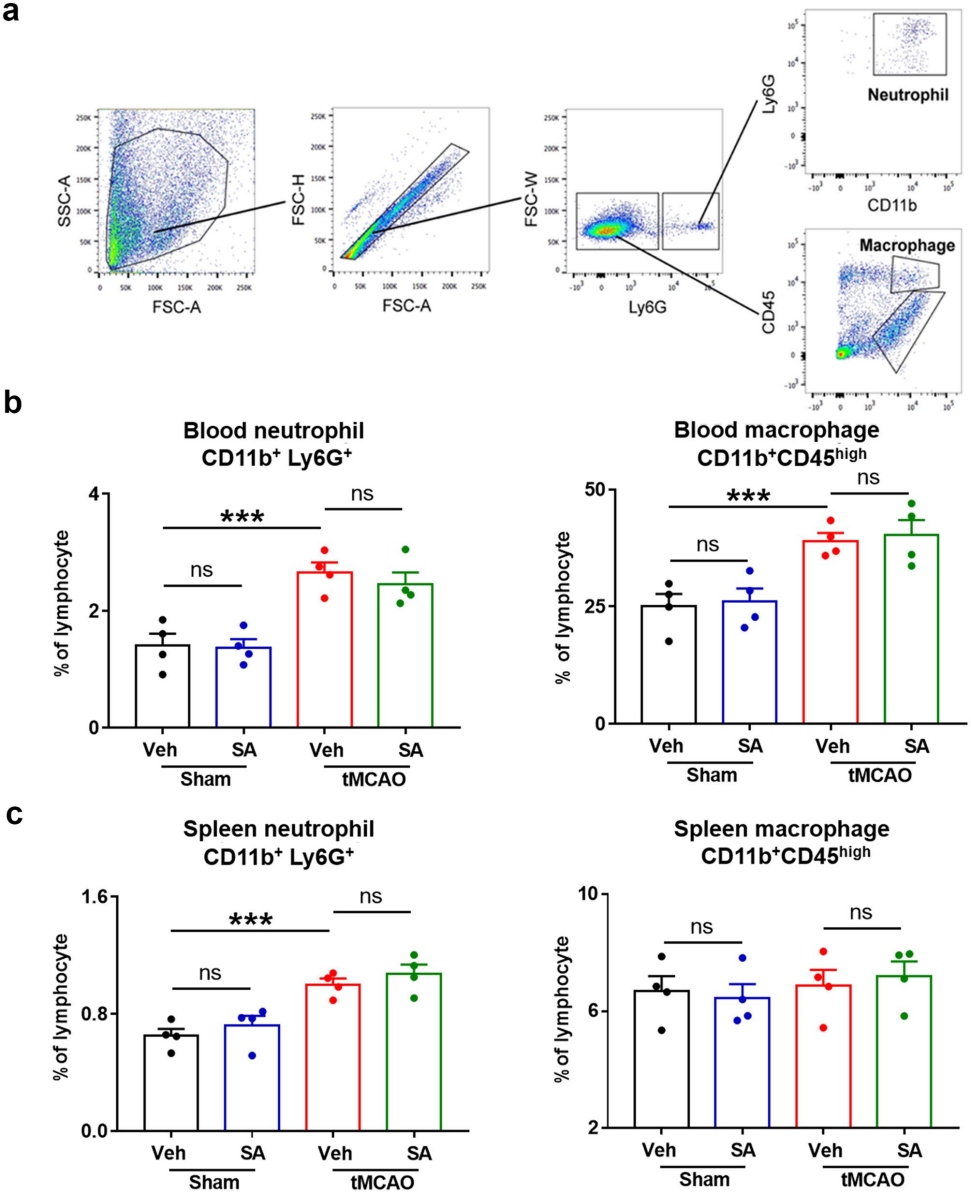
SA administration did not change the number of neutrophils and macrophages in the peripheral system after tMCAO. **a** Representative FACS analyses of neutrophils (CD11b + Ly6G +) and macrophages (CD11b + Ly6G- CD45high). **b-c** Neutrophils and macrophages were quantified and presented as the number of cells per 103 single cells in blood **b** or spleen **c** n = 4 mice/group. All data are presented as mean ± SEM. **p ≤ 0.05, **p ≤ 0.01, ns: not significant, as indicated. SA: salvinorin A; tMCAO, transient Middle Cerebral Artery Occlusion

To verify whether SA treatment decreased the infiltration of immune cells into the brain by suppressing peripheral inflammation, we tested the expression of immune cells in the blood and in spleen tissue to explore changes in the peripheral immune system after tMCAO (Fig. 4 and Fig. S1). The flow cytometry-based strategy results established that tMCAO significantly increased the neutrophils and macrophage percentages both in the blood (Fig. 4a-b) and spleen (Fig. 4c). The percentage of blood Treg also increased after tMCAO (Fig. S1a-b). SA administration did not affect the percentages of peripheral neutrophils and macrophage after tMCAO in mice (Fig. 4a-c). It suggests that SA treatment did not suppress peripheral inflammation after tMCAO.

### SA Treatment had a Protective Effect on the Integrity of BBB after tMCAO

**Fig. 5:**
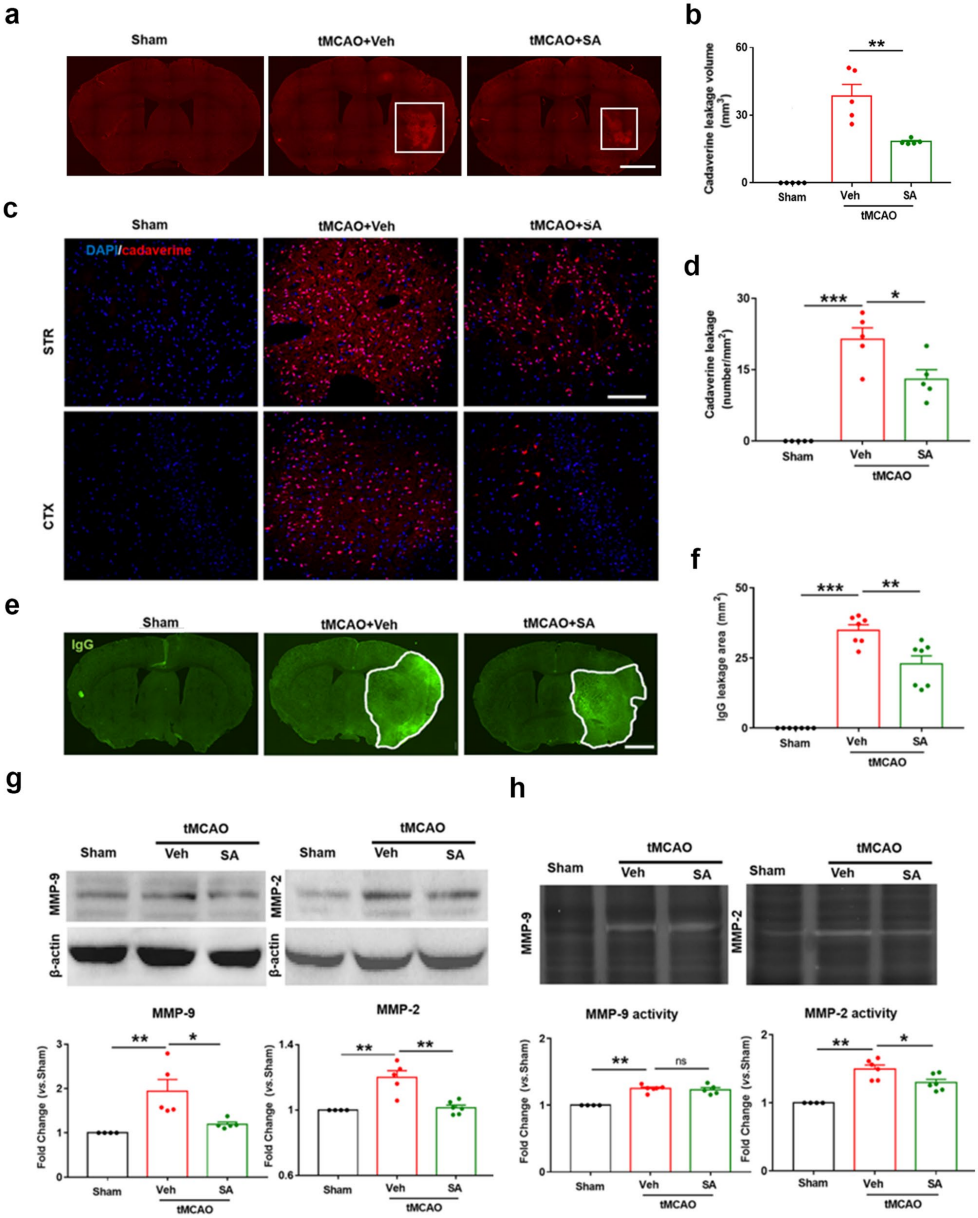
SA treatment had a protective effect on the integrity of BBB after tMCAO. **a-b** Representative images and quantification of the extravasation volume of Alexa 555 cadaverine into the brain parenchyma after tMCAO. n = 5 mice/group (Scale bar: 1 mm.) **c-d** Representative images and quantification of the extravasation density of Alexa 555 cadaverine into the brain parenchyma after tMCAO. n = 5/group (Scale bar: 10 µm.) **e–f** Representative images and quantification of the extravasation area of endogenous plasma IgG into the brain parenchyma after tMCAO. n = 7 mice/group (Scale bar: 1 mm.) **g** MMP-9 or MMP-2 expression levels were measured by western blot in brain tissues, n = 4–6 mice/group. **h** MMP-9 or MMP-2 activity were measured by gelatin zymography in brain tissues. n = 4–6 mice/group. All data are presented as mean ± SEM. *p ≤ 0.05, **p ≤ 0.01, ***p ≤ 0.001, *ns*: no significant, as indicated

We have demonstrated that SA treatment reduced the infiltration of peripheral immune cells into the brain. To confirm that this protective effect is related to the early protection of BBB integrity, we injected cadaverine (0.95KDa, Alexa 555 red) into the femoral vein 23 h after tMCAO and 1 h later perfused the brain with normal saline to take frozen sections of the brain. As the BBB was damaged after tMCAO, the small molecule cadaverine would leaks to the brain tissue. The more serious the damage to the BBB was, the more cadaverine leakage into the brain. The fluorescence of cadaverine leakage was observed and the result showed that the SA treatment group was significantly smaller than the vehicle group at 24 h after tMCAO (Fig. 5a-b). Further observation showed that cadaverine leakage density surrounding the infarct was significantly decreased in the SA treatment group compared to the vehicle group (Fig. 5c-d). Endogenous plasma-derived IgG molecules leaked into the injury area when the BBB was damaged. The IgG immunostaining at 24 h after tMCAO confirmed the above results (Fig. 5e-f).

Matrix metalloproteinases (MMP2 and MMP9) play essential roles in the degradation of the extracellular matrix and in BBB disruption (Rempe et al. 2016). Brain injury tissue from mice at 24 h after tMCAO was subjected to western blots and zymography. Western blot showed that the expression of MMP2 and MMP9 in tMCAO mice was significantly higher than that in the sham group, while the content of MMP2 and MMP9 in the SA treatment group was significantly decreased compared to that in the tMCAO + Veh group (Fig. 5g). The zymography test was employed to detect the activity of MMP2 and MMP9. The experimental results established that tMCAO dramatically increased the activity of MMP2 and MMP9 in mice compared to the sham group. The MMP2 activity was significantly inhibited by SA treatment, while the activity of MMP9 did not significantly change compared to the tMCAO + Veh group (Fig. 5h). Taken together, these data suggested that SA treatment effectively inhibits the expression of MMP2/9 in the brain, and attenuates BBB damage.

### SA Treatment Improved Long-term Stroke Outcomes

We have demonstrated that SA treatment reduces inflammatory factor infiltration into the brain, alleviates BBB disruption and confers robust and long-term preservation of WM. We then proceeded to determine whether administration of SA after tMCAO improves long-term behavioral stroke outcomes.

**Fig. 6:**
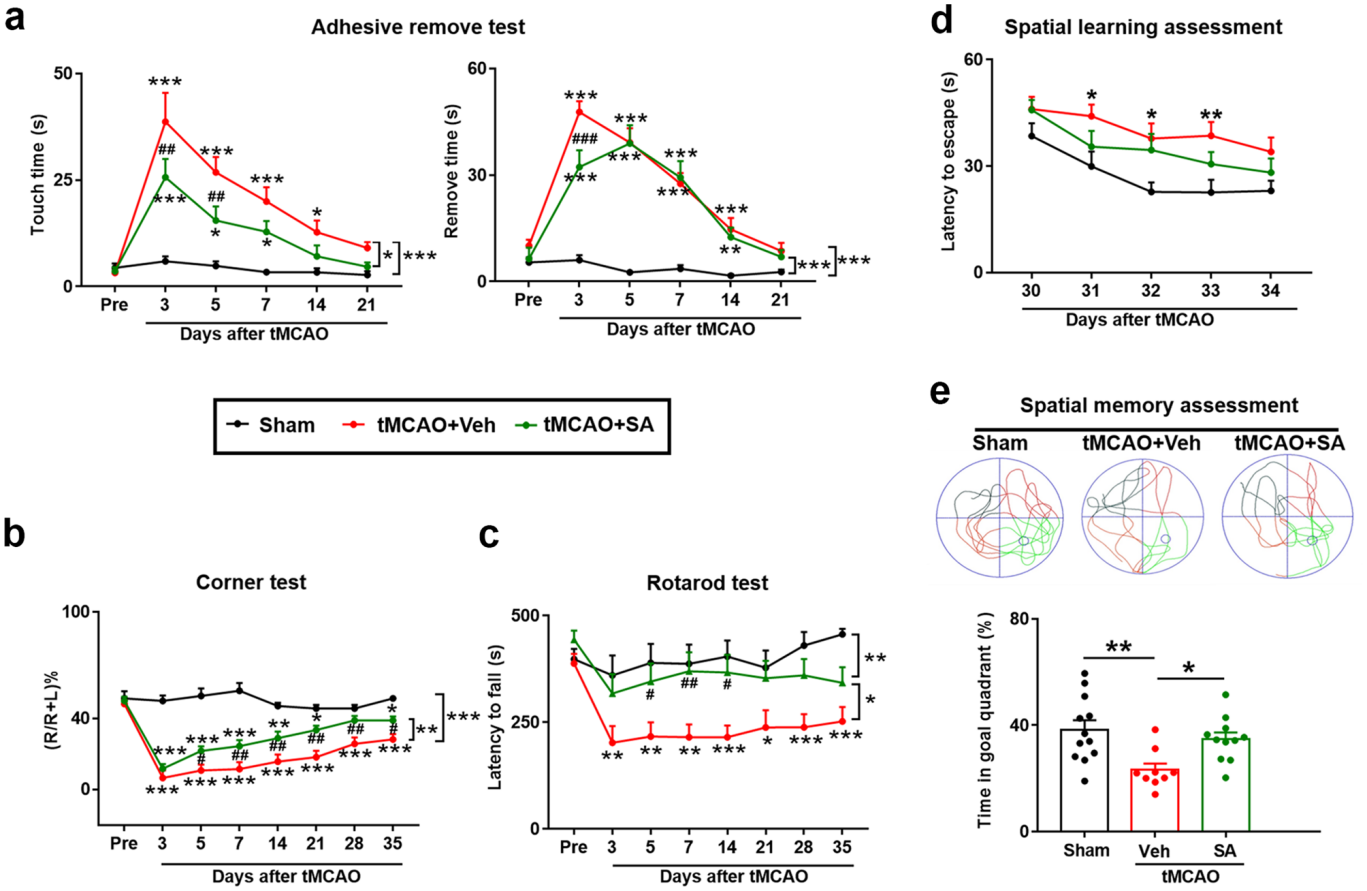
SA treatment improves long-term stroke outcomes. **a–c** Sensorimotor deficits were assessed before (Pre) and up to 35d after tMCAO by adhesive remove test **a**, corner test **b**, and rotarod test **c**. n = 9–12/group. (**d-e**) Water maze was employed to assess the spatial learning **d** and spatial memory **e** at 30–35 days after tMCAO. n = 9–12 mice/group. All data are presented as mean ± SEM. *p ≤ 0.05, **p ≤ 0.01, ***p ≤ 0.001, *vs* Sham, or as indicated; ^#^*p* ≤ 0.05, ^##^*p* ≤ 0.01, ^###^*p* ≤ 0.001, *vs* tMCAO + Veh

A battery of neurobehavioral tests was performed to comprehensively evaluate sensorimotor functions after stroke in the following groups: sham, tMCAO + Veh and tMCAO + SA group, before and up to 35 days after tMCAO. As can be seen from the results, SA administration in sham mice did not induce any change of neurobehavioral or cognitive function (Fig. S2). tMCAO induced significant sensory deficiency and postural asymmetries (adhesive removal test-touch time and corner tests) and motor deficiencies (rotarod and adhesive removal test-removal time) in mice, SA treatment significantly decreased sensorimotor deficits compared with the tMCAO + Veh group (Fig. 6a-c). SA treatment significantly decreased the touch time of adhesive-removal compared to Veh group mice at 3 days after tMCAO, as well as decreased the removal time at both 3 days and 5 days after tMCAO (Fig. 6a). In the rotarod test, SA treatment after ischemic stroke significantly increased the continuous walking time on the rotating rod in mice at 5, 7, and 14 days after the tMCAO, which suggests that SA improved the exercise tolerance decline caused by tMCAO (Fig. 6c). In the corner test, the number of right-turns in the Veh group decreased significantly after tMCAO, indicating that the tactile sensation of the right side of the head was impaired. The number of right-turns in the SA group was significantly increased over that of the Veh group, especially at 7 to 21 days after tMCAO (Fig. 6b). This suggested that the sensory function of the mice recovered faster and better in the SA treatment group than that of the Veh group. Our data demonstrates that SA treatment significantly improved the sensory function and fine motor function of the forelimb after tMCAO.

To further study the effect of SA treatment on cognitive function after ischemic stroke, we performed the Morris water maze which started at 30 days after tMCAO. The learning and training test results are from 30 to 34 days after ischemic stroke. The data demonstrated that the tMCAO + Veh group, but not SA treatment group, had significantly impaired learning function. Though SA treatment dropped the time to find the platform for tMCAO mice, there was no significant statistical difference in the two groups (Fig. 6d). At 35 days after tMCAO, we removed the platform to measure the spatial memory of the mice, which was quantified by the time spent in the target quadrant (the quadrant where the platform was previously located). The time of the tMCAO + Veh group in the target quadrant was significantly lower than that of the sham group, and the SA administration significantly increased the time spent in the target quadrant than tMCAO + Veh group (Fig. 6e). Our results suggest that SA treatment effectively improved cognitive function recovery (especially spatial memory ability) after tMCAO.

### SA Treatment Reduced Brain Tissue Atrophy and Myelin Loss in the Long-term after Ischemic Stroke

**Fig. 7:**
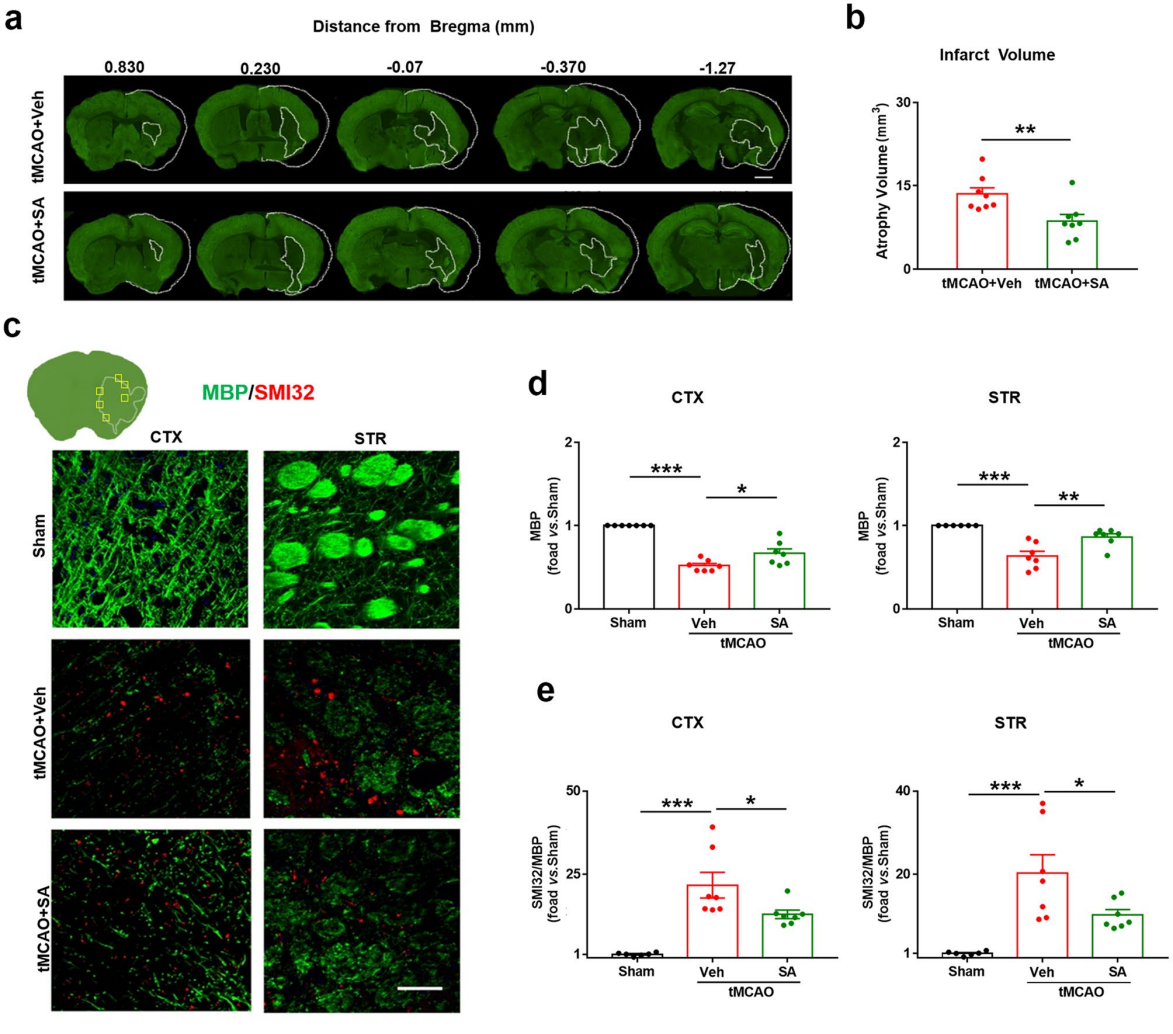
SA treatment reduced brain tissue atrophy and myelin loss in long-term after ischemic stroke. **a-b** Tissue atrophy was measured at 35 d after tMCAO on MAP2 (green)-stained coronal sections. Dashed line, brain infarct. n = 8 mice/group (Scale bar: 1 mm). **c** Representative images of double immunostaining for SMI-32(red) and MBP (green) in the ipsilateral CTX and STR at 35 d after tMCAO. Scale bar: 40 µm. **d-e** Quantification of WM injury, expressed as the fold increases of fold decreases of MBP **d**, and the ratio of SMI-32 to MBP **e**. n = 7 mice/group. All data are presented as mean ± SEM. *p ≤ 0.05, **p ≤ 0.01, ***p ≤ 0.001, as indicated

Brain tissue atrophy directly reflects the degree of stroke injury. To explore the long-term effects of SA treatment on ischemic stroke, brain tissue atrophy was measured at 35 days after tMCAO on MAP2 (green)-stained coronal sections. The present findings showed that brain atrophy was significantly reduced in the tMCAO + SA group mice compared to that in the tMCAO + Veh group mice (Fig. 7a-b). Ischemic stroke elicits profound white matter injury, which plays a key role in the functional recovery and post-stroke rehabilitation (Wang et al. 2016). To verify the long-term preservation by SA treatment on white matter following tMCAO, we examined the axons and myelin sheath damage in the cerebral cortex and striatum (CTX and STR; Fig. 7c-e) by assessing the loss of myelin basic protein (MBP) and the increase in abnormally dephosphorylated neurofilament protein (detected by the SMI-32 antibody). As expected, tMCAO induced obvious axon and myelin sheath damage in mice, which is significantly reduced by SA treatment both in the CTX and STR area of the mouse brain. SA treatment also dramatically reduced the ratio of SMI-32 to MBP at 35 d post-tMCAO, suggesting long-term preservation (or remyelination) of myelinated axons.

## Discussion

In this study, we tested the alteration of immune cells in the peripheral system and the brain after repeated intranasal administration of SA in the acute phase of ischemic stroke. We found that SA reduced the infiltration of peripheral neutrophils and macrophages into brain parenchyma, inhibited the pro-inflammatory polarization of microglia/macrophages and protected the integrity of the BBB after ischemic stroke. In addition, SA reduced white matter damage, and promoted long-term neurological and cognitive function outcome in mice. These data suggest that SA could potentially protect and improve neurological outcome after ischemic stroke via inflammation mediation and BBB protection.

The immune response is one of the main factors affecting stroke pathobiology and long-term prognosis of ischemic stroke (Anrather and Iadecola 2016; Zhang et al. 2019b). It has been demonstrated that the opioid system modulates immune function (Liang et al. 2016). Inflammatory factors include pro-inflammatory factors (IL-1β, TNF-α, TNF- β, iNOS, etc.) and anti-inflammatory factors (TGF- β, IL10, etc.) (Clausen et al. 2008; Lambertsen et al. 2019). Our present findings show that SA treatment after tMCAO significantly decreases the pro-inflammatory factors (IL-1 β, TNF-α, TNF- β, etc.)and Chemokine ligand 2 (CCL2) while increasing the anti-inflammatory factor IL-10.The pro-inflammatory factors (IL-1β, TNF-α, TNF- β, etc.) promote neuronal death and exacerbate the brain infarct while the anti-inflammatory (TGF- β,IL10, etc.) inflammatory cytokines promote the survival of neurons, and inhibit the expression of proinflammatory cytokines, thereby reducing harmful inflammation after ischemic stroke (Ramiro et al. 2018; Yang et al. 2019). In the ischemic penumbra of stroke models, Chemokine ligand 2 (CCL2) levels increase as early as 6 h of reperfusion with peak levels (Dimitrijevic et al. 2007; Guo et al. 2014) with induction of leukocyte recruitment and disruption of BBB integrity (Fang et al. 2018). Moreover, levels of CCL2 are positively correlated with infarct size and enlargement of the ischemic lesion (Fang et al. 2018; Jayaraj et al. 2019). Therefore, the detection of these markers of inflammatory mediators and chemokines can not only detect the overall status of inflammation in the brain, but also indirectly reflect the degree of brain damage after ischemic stroke. Our data is consistent with the effect of SA on inflammatory factors described in previous literature (Aviello et al. 2011).

Our findings also demonstrate that SA administration significantly decreases the infiltration of neutrophils and macrophages into the brain after tMCAO in mice, without altering the cell number of neutrophil and macrophages in the peripheral circulation. Blood-derived neutrophils are crucial leukocytes that infiltrate into the ischemic brain (Cai et al. 2020; Chen et al. 2021).They are detected within 30 min, peaking between days 1–3 after ischemic stroke (Jickling et al. 2015). Neutrophils play a major pathological role in acute ischemic injury resulting in severe endothelial damage, destruction of adjacent blood vessels, and in some cases hemorrhagic transformation (Perez-de-Puig et al. 2015). Previous study reported that initial over activation of the peripheral immune system after stroke might result in the expansion of Tregs which help to suppress the systemic inflammation (Meng et al. 2016; Wang et al. 2021). This is well in line with our experimental result that Tregs significantly increased in the peripheral system of mice that suffered tMCAO. Interestingly, SA administration significantly decreased the Tregs in the blood after tMCAO (Fig. S2). It might be that SA reduced the systemic inflammatory factors instead of immune cells in the early phase after stroke. In the present study, we focused our investigation on the effects of SA on neutrophils and microglia/macrophages, the effects of treatment on the Tregs in the brain and inflammatory factors in the peripheral system after tMCAO warrant further studies.

Suppressing neutrophil/macrophage infiltration and preserving BBB integrity which were confirmed in the study may complement each other. Recruited peripheral immune cells into the injured areas release MMP-9 and MMP-2, leading to secondary BBB damage (Qiu et al. 2021). Circulating neutrophils are the main infiltrating peripheral immune cells within one hour and peaking by 24 h post-injury (Gelderblom et al. 2009). Previous studies demonstrated that neutrophil depletion reduced BBB leakiness after brain injury (Bodnar et al. 2021; Wei et al. 2021). Conversely, the increased permeability of BBB facilitates neutrophil infiltration into the brain after ischemic stroke (Obermeier et al. 2013). In this study, SA treatment significantly decreased the neutrophil/macrophage infiltration into the brain tissue while not altering the peripheral immune cells, suggesting that SA may play an immunomodulatory role though preserving BBB integrity. The cadaverine leakage density and plasma IgG leakage detection further demonstrated the beneficial effect of SA on the BBB. The two important mechanisms, suppressing neutrophil/macrophage infiltration and preserving BBB integrity may both contribute to long-term benefits. Future studies are needed to clarify which mechanism is more important and which occurs first.

Administration of SA is considered to be efficient for neuroprotection in several studies. Some main experimental characteristics are summarized in Table 1. We can conclude that single or multiple, pre-or post-treatment, different time-point and doses treatments in different animal models all show a neuroprotective role of SA, suggesting a potential application for a stroke treatment strategy.

**Table 1:** Summary of experimental characteristics of SA treatment associated studies

Ref	Drug administration time	Animal species/gender	Administration route	Experimental model	SA doses	Experimental results
(Wu et al. 2021)	20 min after stroke	rhesus monkey/male	intranasal	MCAO with an autologous blood clot	25 mg/kg	SA reduced infarct volume and improved neurological outcomes
(Chen et al. 2016)	10 min after initiation of reperfusion	Mouse/male	intranasal	120-min tMCAO	50 µg/kg	SA reduced infarct volume and improved the neurological score, as well as preserved the blood brain barrier at 24 h after tMCAO
(Chen et al. 2014)	immediately before hypoxic insult	neonatal mouse	i.p. injection	hypoxia model	0.5 mg/kg	SA improved neurological outcomes and reduced mortality rate
The present study	10 min after initiation of reperfusion and re-treated once every 2 days for 6 consecutive days	Mouse/male	intranasal	60-min tMCAO	50 µg/kg	SA improves long-term neurological functional via immuno-modulation and preserving blood–brain barrier integrity

There are some limitations in this study. Only male mice were employed for this initial study out of consideration that reproductive hormonal concentration changes might have an effect and complicate the analysis of neurofunctional outcomes in female mice. A previous study demonstrated that ischemic neuroprotection with the selective kappa-opioid receptor agonist (BRL 52537 hydrochloride) is gender specific in rat (Chen et al. 2005). Schmidt et al. indicated that SA distribution and elimination differs among different genders and the kinetics are slower in female than in male monkeys (Schmidt et al. 2005), suggesting the importance of gender specific studies to investigate how SA works in both sexes for the ultimate goal of clinical translation in the future studies. Additional studies are also warranted to distinguish which effect, suppressing neutrophil and macrophage infiltration or preserving BBB integrity, occurs first and which is more important for the long-term benefits of SA administration after stroke.

## Conclusion

In this study, intranasal administration of SA improved long-term neurological function via immuno-modulation and preserving blood–brain barrier integrity in a mouse ischemic stroke model, suggesting that SA could be potentially serve as an innovative alternative treatment strategy for ischemic stroke.

## Supplementary Information

Below is the link to the electronic supplementary material.

Supplementary file1 (DOCX 1786 KB)
