# Supplementary material for: Intranasal Salvinorin A Improves Long-term Neurological Function via Immunomodulation in a Mouse Ischemic Stroke Model
Source: J Neuroimmune Pharmacol. 2021 Oct 1;17(1-2):350–66. doi: 10.1007/s11481-021-10025-4 (PMC9726789; doi:10.1007/s11481-021-10025-4)
Supplement: Supplementary file 1 — Supplementary file1 (DOCX 1786 KB) [file 11481_2021_10025_MOESM1_ESM.docx]

**Supplementary Materials**

1. **Supplementary Figures**

**S1:** **SA administration reduced the Treg cells in the blood but not in spleen and did not change the Breg cells number in the blood or the spleen after tMCAO.**

**
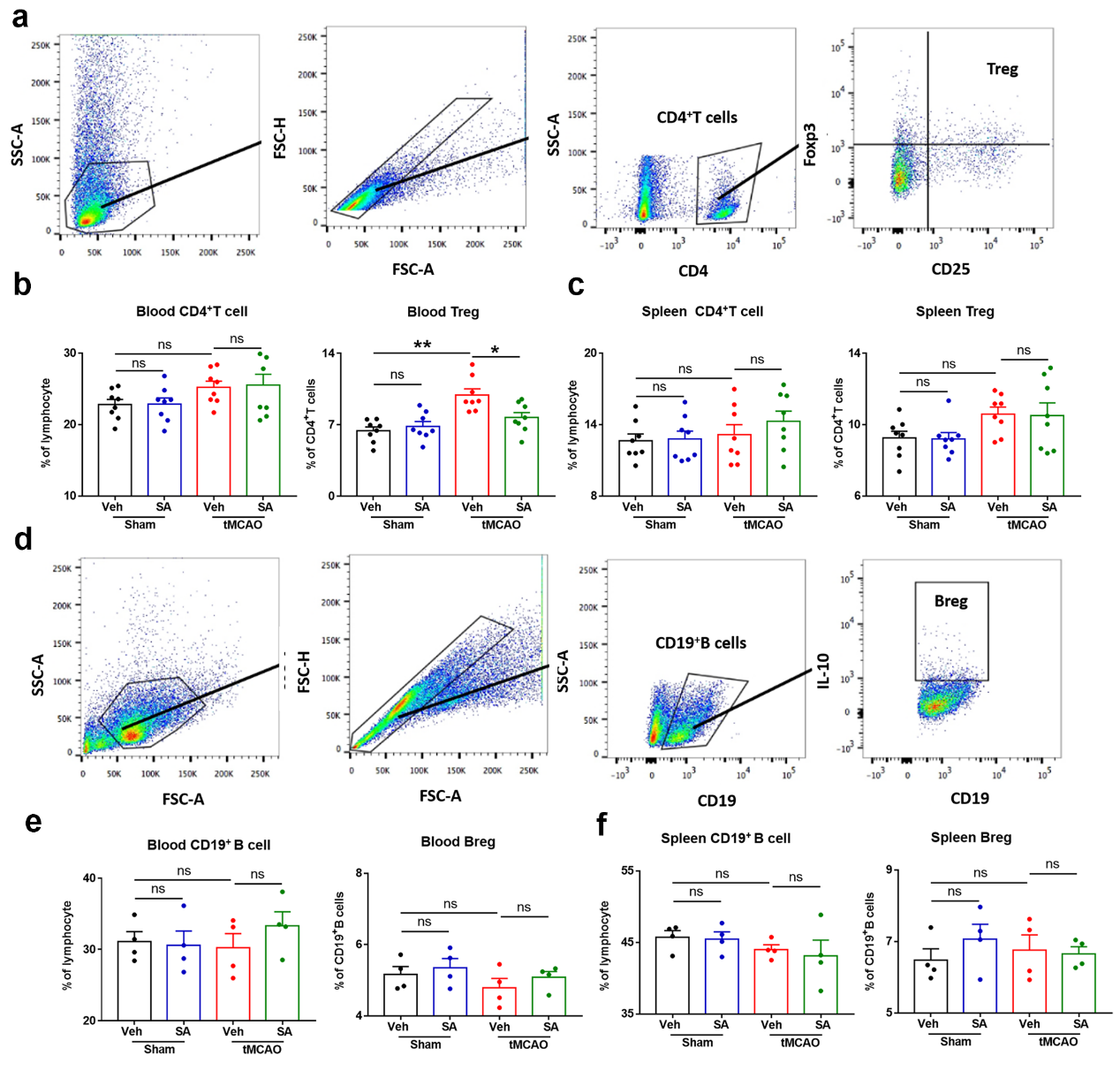
**

**Fig.S1: SA administration reduced the Treg cells in the blood but not in spleen and did not change the Breg cells number in the blood or the spleen after tMCAO.** (a) Representative FACS analyses of CD4^+^ T cell and Tregs in the blood and spleen. (b-c) Quantification of CD4^+^ T cell and Treg cells in the blood (b) and spleen(c). (d) Representative FACS analyses of CD19^+^ B cell and Breg cells in the blood and spleen. (e-f) Quantification of CD19^+^ B cell and Breg cells in the blood (e) and spleen (f). n=4 mice/group. All data are presented as mean ± SEM. ***p*≤0.05, ***p*≤0.01, *ns*: not significant, as indicated. SA: salvinorin A; tMCAO, transient Middle Cerebral Artery Occlusion.

**S2: SA administration did not alter neurobehavioral and cognitive function in sham mice.**

**
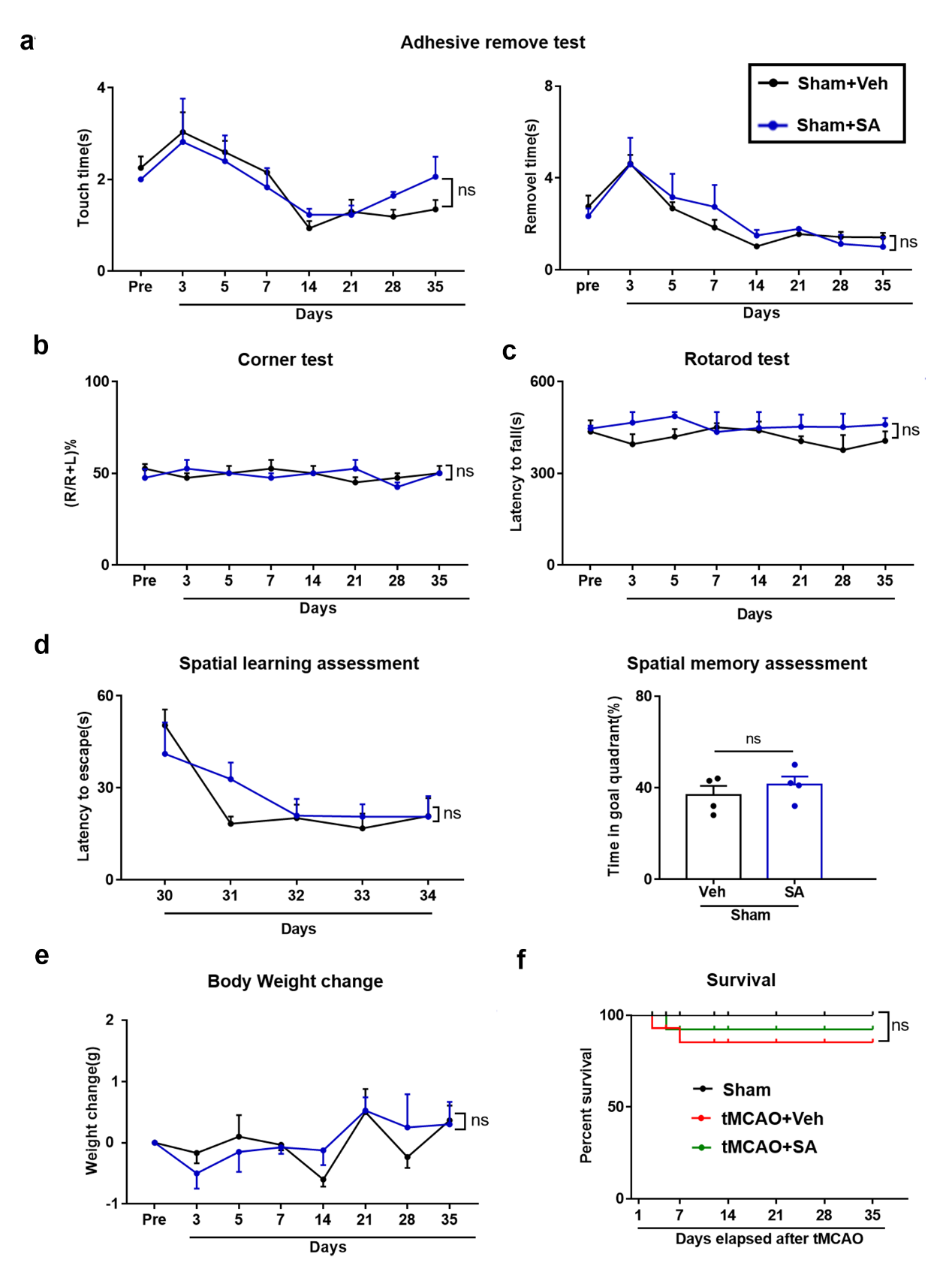
**

**Fig.S2: SA administration does not change neurobehavioral and cognitive function in sham mice.** (a–c) Sensorimotor deficits were assessed before (Pre) and up to 35d after SA administration in normal mice by (a) Adhesive remove, (b) Corner test, and (c) Rotarod test n=6/group. (d) Water maze was employed to explore the learning time and memory at 30-35 days after SA administration. n=4/group. (e) Body weight record before (Pre) and up to 35d after SA administration. (f) Percent of survival in indicated days. n=14 mice /group. All data are presented as mean ± SEM. **p* < 0.05, *ns*: not significant, as indicated. SA: salvinorin A; tMCAO, transient Middle Cerebral Artery Occlusion.

1. **Primers for qPCR**

IL-1β: CACCTCTCAAGCAGAGCACAG-3’ 5’- GGGTTCCATGGTGAAGTCAAC

TNF-α: AAATGGGCTCCCTCTCATCAGTTC-3’; 5’- TCTGCTTGGTGGTTTGCTACGAC

TNF-β: TCCACTCCCTCAGAAGCACT-3’; 5’-GAAAAGAGCTGGACCTCGTG;

iNOS: CAAGCACCTTGGAAGAGGAG-3’; 5’- AAGGCCAAACACAGCATACC

IL-10: CTTACTGACTGGCATGAGGATCA-, - GCAGCTGTAGGAGCATGTGG

CCL-2: CAGCCAGATGCAATCAATGCC-3’; 5’-TGGAATCCTGAACCCACTTCT

CCL-3: TGTACCATGACATCTGCAAC-, -CAACGATGAATTGGCGTGGAA

**3. Flow Cytometry**

The single cell suspension of brains were prepared from the ischemic hemispheres using the Neural Tissue Dissociation Kit (Miltenyi Biotec). After digestion and 70μm-filter filtration, the cell suspension of brain was prepared using a 30%-70% Percoll (GE Healthcare BioSciences, USA) gradient centrifugation to isolate myelin debris and red blood cells. After centrifugation, cells at the interface of 30% and 70% Percoll were collected then washed for the subsequent staining procedure. The spleen was ground and diluted with Hank's Balanced Salt Solution (HBSS). The cell suspension of spleen was filtered with a 70μm filter, then the red blood cells were lysed with ACK lysis buffer (Thermo Fisher Gibco, USA). For blood, Ficoll (GE Healthcare BioSciences, USA) gradient centrifugation was used to isolate red blood cells.

For all staining procedures, CD16/32 antibody (1:100, CAT#48-0451-82, Thermo Fisher eBioscience, USA) was used for blocking by 10 minutes on ice, followed by 30-minutes of fluorophore-labeled antibodies on ice in the dark. For intracellular staining, after surface antigen antibody staining, the cells were permeabilized and fixed with the Cytofix/Cytoperm Fixation/Permeablization Kit (BD Biosciences, USA) or Foxp3 / Transcription Factor Staining Buffer (Thermo Fisher eBioscience, USA), and then stained with fluorophore-labeled antibodies for 30 min on ice in the dark.

Anti-mouse antibodies used for this study included: CD45-eFluor 450 (1:100, CAT#48-0451-82, Thermo Fisher eBioscience, USA), CD11b-APC cy7 (1:400, CAT#47-0112-82, Thermo Fisher eBioscience, USA), Ly6G-PE (1:400, CAT#12-9669-82, Thermo Fisher eBioscience, USA), CD11c-PerCP cy5.5 (1:200, CAT#45-0114-82, Thermo Fisher eBioscience, USA), CD19-FITC (1:200, CAT#11-0193-82, Thermo Fisher eBioscience, USA), CD4-FITC (1:400, CAT#11-0042-82, Thermo Fisher eBioscience, USA), CD25-Alexa Fluor 700 (1:400, CAT#56-0251-82, Thermo Fisher eBioscience, USA), IL-10-PE (1:400, CAT#554467, BD Biosciences, USA ), and Foxp3-PE(1:100, CAT#563101, BD Biosciences, USA). Flow cytometry was performed using the BD LSRFortessaTM Flow Cytometer (BD Biosciences, USA). Data analysis was performed using FlowJo software (FlowJo, LLC).

**4. Neurobehavoral assessments**

**Corner test**

The corner test was used for detecting long-term sensorimotor dysfunction after tMCAO(Zhang et al., 2002; Shi et al., 2017). The pre-training was performed for 3 consecutive days before tMCAO. The mouse was placed between two boards that were attached at a 30° angle with a small opening along the joint and were encouraged to enter into the corner. When entering deep into the corner both sides of the vibrissae are stimulated together. The non-ischemic (sham control) mouse turns either left or right, but the ischemic mouse preferentially turns toward the non-impaired, ipsilateral (right) side. The number of turns towards the right or left direction were recorded for ten trials for each test. The frequency of right turns was calculated and presented as (R/R+L) % (R, number of right turns; L, number of left turns).

**Rotarod test**

The rotarod test was used to assess motor performance by measuring the time to remain on an elevated rotating accelerating rod (4 to 40 r/min over 300 s) (Shi et al., 2016). Mice were trained for three trials per day for three consecutive days before the surgery. The average time of the three trials during the last day of training was recorded as the pre-surgery baseline value. After surgery, three acclimation trials were performed in 5 minutes with a 15 minutes rest interval for physical recovery, and the data for 3 trials were used to calculate the mean latency to fall on that day.

**Adhesive removal test**

The adhesive removal test was performed to evaluate sensory and motor deficits(Bouet et al., 2009). The mice were trained by performing 1 trial per day for 3 days before surgery. After a habituation period of 15 minutes in the testing box, adhesive tape (3X4 mm^2^) strips were applied with equal pressure on each animal paw. The animal's behavior is observed, and the time to beginning to contact the tape and the time till removal of the tape is recorded.

**Morris water maze**

The Morris water maze test was performed to assess spatial learning and memory. The pre-training was performed for 3 consecutive days before tMCAO. The swimming time until the mouse located the platform is recorded from day 30 to day 34 after tMCAO, as previously described (Zhang et al., 2019). If the mouse located the platform in 60 s, it was immediately removed from the pool. If the mouse failed to locate on the platform after 60 s of swimming, the mouse was gently guided to the platform and allowed an additional 15 s for reorientation. The platform was removed from the pool to examine spatial reference memory at 35 days after tMCAO. The mice are allowed to swim freely for 60s.The movement in the water is monitored by an overhead camera and recording of the movement trajectories, time across quadrants, swimming speed and other indicators are performed.

**References**

Bouet V, Boulouard M, Toutain J, Divoux D, Bernaudin M, Schumann-Bard P, Freret T (2009) The adhesive removal test: a sensitive method to assess sensorimotor deficits in mice. Nat Protoc 4:1560-1564.

Shi Y, Jiang X, Zhang L, Pu H, Hu X, Zhang W, Cai W, Gao Y, Leak RK, Keep RF, Bennett MV, Chen J (2017) Endothelium-targeted overexpression of heat shock protein 27 ameliorates blood-brain barrier disruption after ischemic brain injury. Proceedings of the National Academy of Sciences of the United States of America 114:E1243-e1252.

Shi Y, Zhang L, Pu H, Mao L, Hu X, Jiang X, Xu N, Stetler RA, Zhang F, Liu X, Leak RK, Keep RF, Ji X, Chen J (2016) Rapid endothelial cytoskeletal reorganization enables early blood-brain barrier disruption and long-term ischaemic reperfusion brain injury. Nat Commun 7:10523.

Zhang J, Zhang W, Gao X, Zhao Y, Chen D, Xu N, Pu H, Stetler RA, Gao Y (2019) Preconditioning with partial caloric restriction confers long-term protection against grey and white matter injury after transient focal ischemia. Journal of cerebral blood flow and metabolism : official journal of the International Society of Cerebral Blood Flow and Metabolism 39:1394-1409.

Zhang L, Schallert T, Zhang ZG, Jiang Q, Arniego P, Li Q, Lu M, Chopp M (2002) A test for detecting long-term sensorimotor dysfunction in the mouse after focal cerebral ischemia. Journal of neuroscience methods 117:207-214.
